# Effects of *Daqu* Attributes on Distribution and Assembly Patterns of Microbial Communities and Their Metabolic Function of Artificial Pit Mud

**DOI:** 10.3390/foods11182922

**Published:** 2022-09-19

**Authors:** Yu Mu, Jun Huang, Rongqing Zhou, Suyi Zhang, Hui Qin, Hanlan Tang, Qianglin Pan, Huifang Tang

**Affiliations:** 1 College of Biomass Science and Engineering, Sichuan University, Chengdu 610065, China; 2 National Engineering Research Center of Solid-State Manufacturing, Luzhou 646000, China; 3 Luzhou Laojiao Co., Ltd., Luzhou 646699, China

**Keywords:** artificial pit mud, fortified *Daqu*, community assembly, co-occurrence network, metabolic function

## Abstract

*Daqu* provides functional microbiota and various nutrients for artificial pit mud (APM) cultivation. However, little is known about whether its attributes affect the microbiome and metabolome of APM. Here, two types of APM were manufactured by adding fortified *Daqu* (FD) and conventional *Daqu* (CD); they were comprehensively compared by polyphasic detection methods after being used for two years. The results showed that FD altered the prokaryotic communities rather than the fungal ones, resulting in increased archaea and *Clostridium_sensu_stricto*_12 and decreased eubacteria and *Lactobacillus*. Correlation analysis suggested that these variations in community structure promoted the formation of hexanoic acid, butyric acid, and the corresponding ethyl esters, whereas they inhibited that of lactic acid and ethyl lactate and thus improved the flavor quality of the APM. Notably, pH was the main driving factor for the bacterial community variation, and the total acid mediated the balance between the stochastic and the deterministic processes. Furthermore, the results of the network analysis and PICRUSt2 indicated that FD also enhanced the modularity and robustness of the co-occurrence network and the abundance of enzymes related to hexanoic acid and butyric acid production. Our study highlights the importance of *Daqu* attributes in APM cultivation, which are of great significance for the production of high-quality strong-flavor Baijiu.

## 1. Introduction

Baijiu not only has a long history but also has attributes of the traditional Chinese culture [1]. Among the 12 main categories, strong-flavor Baijiu (SFB) is the most popular and occupies about 70% of the Baijiu market in China [2]. One of the unique characteristics of SFB production is the solid-state fermentation process carried out anaerobically in an underground mud pit, which is a rectangular pit (approximately: 2 m × 3 m × 2 m) lined by the special fermented clay called pit mud (PM) [3]. PM is an important sustained-release source of the functional microbial consortia for SFB fermentation and plays an essential role in the synthesis of hexanoic acid and butyric acid [4,5]. These two acids can be esterified with ethanol to further produce ethyl hexanoate and ethyl butyrate, the feature flavor compounds of SFB, resulting in an improved flavor quality of the fermented grains (referred to as *Zaopei*) and fresh Baijiu [6,7]. However, the natural evolution of PM microbiota is very slow during the brewing process and might need more than 20 years of domestication uninterruptedly to produce high-quality SFB [8]. Therefore, it has become one of the focuses of the development of the artificial PM (APM) manufacturing technology since the 1960s [3].

APM manufacturing is a directional evolution process of functional consortia under anaerobic conditions; yellow and black clays and peats are the main raw materials, while high-quality PM, functional strain, *Huangshui* and *Daqu* are the primary microbial sources [9,10,11]. Traditionally, the main focus of APM improvement studies has been on the different formulas, the functional strains, and the cultivation patterns and times due to the scarcity of high-quality PM resources and the frequent degradation of aged PM [10,11,12]. However, the effects of *Daqu* on APM quality have received little attention, despite the fact that it can provide the functional microorganisms, enzymes, and nutrients for APM cultivation [10,11,13]. Notably, more than 30% of the bacterial communities, composed of massive aerobes/facultative-aerobes and a few anaerobes in *Daqu* shared in both *Zaopei* and the PM [5], and enormous fungi from *Daqu* also helped to maintain the fungal diversity in the PM for promoting community development [14]. In addition, the interphase mass transfer in the SFB brewing micro-ecosystem affects not only material metabolism but also interspecies interactions [15,16] and was closely related to the *Daqu* attributes. For example, the contents of hexanoic acid and ethyl hexanoate in *Zaopei* and fresh Baijiu were increased by regulating the interactions among the functional consortia when the fortified *Daqu* (FD) inoculated with *Bacillus* was used [17,18]. Meanwhile, it was also conducive to enhancing the abundance of hexanoic acid-producing bacteria and methanogenic archaea in PM after continuous use in multiple batches [19,20]. This might be related to the antagonism between *Bacillus* and *Lactobacillus* frequently observed in Baijiu brewing micro-ecosystems [21,22,23]. *Bacillus* was symbiotic with several members of *Clostridia* in PM and contributed to the synthesis of hexanoic acid [9,24], while the high abundance of *Lactobacillus* inhibited the growth of methanogens and consequently their interspecies hydrogen transfer with *Clostridium* [25,26]. Therefore, we speculated that FD may also be conducive to the promotion of the directional evolution of the APM microbial consortia, revealing that its underlying mechanism is of great significance for the production of high-quality SFB.

In the present work, two types of APMs were manufactured and investigated through an in situ experiment to explore the impact of FD on the community evolution of the APM. After being used for SFB fermentation for two years, we compared the core microbes and community structures via fluorescence in situ hybridization (FISH) and high-throughput sequencing. Meanwhile, the metabolic profiles, the physicochemical properties, and their correlation with the microbial communities were analyzed. Furthermore, the differences in community assembly pattern, co-occurrence network, and metabolic function were also evaluated to deepen our knowledge regarding the potential mechanism of FD improving the quality of APM. This study promotes the understanding of the directional evolution of microbial consortia and provides new insights for improving the quality and metabolic activity of APM.

## 2. Materials and Methods

### 2.1. The Manufacture of Fortified Daqu

The manufacture of FD was carried out in Luzhou Lao Jiao Co., Ltd. (Luzhou, Sichuan, China) according to our prior work [27]. Briefly, *B. velezensis* with a high yield of tetramethylpyrazine and *B. subtilis* with a high yield of 4-ethylguaiacol were inoculated into the water as the starter at the ratio of 1:1 after being cultured (37 °C, 24 h), and the final concentration of the starter was 2.3 × 10^6^ CFU/mL. Then, the starter was mixed with crushed wheat to shape the *Daqu* brick (30 cm × 20 cm × 7 cm), which had a moisture content of 34%~36%. The CD was produced in the same batch without inoculating the starter. The FD and CD were collected after storage for three months, and the differences in the enzymatic activity, microbial community, and volatile compounds between them were examined [27].

### 2.2. The Artificial Pit Mud Manufacturing and Sample Collection

The APM manufacturing process is described in our previous study [11], with slight modifications. Briefly, dried and smashed yellow clays were mixed with black clays and peats at a ratio of 10:3:1 (*w*/*w*/*w*) to form the solid matrix. Then, the PM culture liquid was mixed with tap water, tail liquor, and *Huangshui* (a by-product of SFB fermentation) at a ratio of 1:10:5:1.5 (*v*/*v*/*v*/*v*) to form the liquid matrix. After the *Daqu* was added at 5% by weight of the solid matrix, the solid and liquid matrixes were evenly mixed at 1: 2 (*w*/*v*). Finally, the mixture was piled outside, covered with polyester films and incubated at an ambient temperature for 60 days. The PM culture liquid was obtained by inoculating 1% of the aged PM (>50 years) into the semisynthetic medium and anaerobic fermentation for 20 days. The medium was composed of 5 g/L NaHCO_3_, 1 g/L yeast extract, 1 g/L peptone, 3 g/L glucose, 0.2 g/L cysteine hydrochloride, 170 mL/L salt solution A (3 g/L KH_2_PO_4_, 6 g/L NaCl, 3 g/L (NH_4_)_2_SO_4_, 0.3 g/L CaCl_2_, 0.3 g/L MgSO_4_), 170 mL/L salt solution B (3 g/L K_2_HPO_4_), and 150 mL/L *Huangshui*.

The fortified APM (FPM) and conventional APM (CPM) were manufactured by adding the FD and CD, respectively, and were further used to construct pits in Luzhou Lao Jiao Co., Ltd. (Luzhou, Sichuan, China). Then, these pits were used for SFB fermentation with the identified process parameter, raw material, and starter in the same workshop. After two years, five pits lined with FPM and CPM, respectively, were randomly selected and a five-point sampling method was used to take 100 g PM from the center and four corners of the bottom surface (depth: 2 cm). After that, these five subsamples from each pit were mixed uniformly as one biological sample, and a total of 10 biological samples (5 CPM + 5 FPM) were obtained in this study. Each biological sample was divided into two parts. One was stored at −80 °C for the analysis of the microbial communities, and another was stored at 4 °C for the detection of the physicochemical properties and metabolites.

### 2.3. Analysis of Microbial Community

#### 2.3.1. Fluorescence In Situ Hybridization (FISH)

The 1.00 g APM and 25 mL sterilized PBS buffer (10 mmol/L, pH 7.2) were accurately added into a 50 mL sterilized centrifuge tube and vortex mixed for 5 min. Then, the pooled supernatant was obtained after centrifugation (800 r/min, 4 °C, 10 min) thrice and further centrifuged (12,000 r/min, 4 °C, 10 min) to collect the precipitate, which was stored at −20 °C after being washed thrice with the PBS buffer. According to the conditions and procedures described by our previous study [17], FISH was used to detect the composition of the core microbes through several targeted probes. All probes were synthesized with the dye Cy3 at the 5′ end by Sangon (Shanghai, China), and the detailed information is shown in Table S1.

#### 2.3.2. High-Throughput Sequencing

The total DNA of the APM sample was extracted using the Fast DNA SPIN extraction kit (MP Biomedicals, Santa Ana, CA, USA), according to the manufacturer’s protocols. The quality and quantity of DNA were assessed by 0.8% (*w*/*v*) agarose gel electrophoresis and NanoDrop ND-1000 spectrophotometer (Thermo Scientific, Waltham, MA, USA). In accordance with our previous study [19], 338F/806R and ITS5F/ITS1R primers were used for the PCR amplification of the bacterial 16S rRNA V3-V4 region and the fungal ITS1 region, respectively, and then, high-throughput sequencing (PE, 2 × 250 bp) of the PCR products was performed at Shanghai Personal Biotechnology Co., Ltd. (Shanghai, China).

The raw sequencing data were processed by QIIME2 (2019.4). After removing the primers from the raw sequences, the remaining sequences were used to generate an amplicon sequence variant (ASV) table by DADA2, which includes quality filtering, denoising, merging, and chimera removing [28]. Subsequently, all the ASVs were assigned using the naive Bayes taxonomy classifier in the feature-classifier plugin against the Silva (v 132) and UNITE (v 8.0) databases. All the raw sequences were deposited in the NCBI Sequence Read Archive under accession number PRJNA835955.

### 2.4. Analysis of Metabolites

#### 2.4.1. Analysis of Organic Acids

An Agilent 1260 HPLC system equipped with an Alltech OA-1000 organic acid column (300 × 7.8 mm, Agilent, SC, USA) was used to analyze the organic acids [17]. Briefly, 5.00 g APM was mixed with 20 mL mobile phase and the mixture was extracted by ultrasonic treatment for 60 min; then, the supernatant was collected after centrifugation at 12,000 r/min at 4 °C for 10 min. The supernatant was purified by an activated SPE C18 column and then filtered by a 0.22 μm filter membrane. The HPLC conditions were as follows: injection volume, 20 μL; mobile phase, 9 mmol/L H_2_SO_4_ solution; flow rate, 0.6 mL/min; column temperature, 75 °C; detection wavelength, 210 nm. All the samples were measured in triplicate.

#### 2.4.2. Analysis of Volatile Compounds

Trace 1300-TSQ 9000 GC-MS (Thermo Scientific, Waltham, MA, USA) equipped with a VF-WAX-MS capillary column (30.0 m × 0.25 mm × 0.25 μm, Agilent, Santa Clara, CA, USA) was applied to detect the volatile flavors of the PM [20]. Briefly, 1.00 g APM and 20 μL methyl octanoate (internal standard, 0.0079 g/100 mL) were accurately added to a 20 mL headspace bottle, and a 50/30 μm DVB/CAR/PDMS fiber (2 cm, Supelco, Bellefonte, PA, USA) was used to extract volatile flavors at 60 °C for 50 min, and then, the extraction head was removed and inserted into the inlet and desorbed for 5 min. The GC conditions were as follows: inlet temperature, 270 °C; carrier gas, high-purity helium (>99.999%); flow rate, 1 mL/min; spitless. The temperature of the column was as follows: 40 °C for 5 min, 4 °C/min to 100 °C, and 6 °C/min to 230 °C for 10 min. The MS conditions were as follows: ion source temperature, 250 °C; transmission line temperature, 300 °C; ionization mode, EI (70 eV); scan range, 35–400 amu. After comparing with the NIST 2017 library, only compounds with similarity (SI) >800 remained for further analysis (the highest value is 1000). All the samples were measured in triplicate.

### 2.5. Detection of Physicochemical Properties

The moisture content was detected by drying the samples at 105 °C for 4 h. The fresh PM was mixed with deionized water at a ratio of 1:5 (W/V), and the pH was determined using a pH meter (pHS-3C, INESA, Shanghai, China) after standing for 30 min [8]. Based on standard NaOH (0.1 mol/L) solution, the contents of total acid (TA) and total ester (TE) were determined using the acid-base titration and the titrimetric method after saponification, respectively. The content of ammoniacal nitrogen (NH_4_^+^-N) and the available phosphorus (AP) were measured by the Nessler’s reagent colorimetric and ammonium molybdate methods, respectively [16]. All the samples were measured in triplicate.

### 2.6. Statistical Analysis

All the data in this study were presented as mean ± standard deviation, and all statistical analyses were conducted in R software (v 4.1.3) unless otherwise stated. The significant differences between each physicochemical property, metabolite, and dominant genus were determined by the *t*-test in the “stats” package. The α- and β-diversity were calculated by the “vegan” package and visualized by boxplot and principal coordinate analysis (PCoA). The “metagenomeSeq” package was further applied to determine the difference in bacterial community and visualized by the Manhattan plot. The principal component analysis (PCA) and the cluster heatmap analysis of the volatile flavors were performed by Simca 14.0 and the “pheatmap” package, respectively. The Spearman’s rank correlations between the dominant microbial genera and volatile compounds were conducted using the “Hmisc” package and visualized in Cytoscape (v. 3.60) based on |ρ| > 0.7 and *p* < 0.05. Mantel tests between the overall communities and the physicochemical properties were performed by the “ggcor” package to determine the driving factors of the community variation. The redundancy analysis (RDA) between the dominant bacteria and the physicochemical properties was conducted using the “vegan” package, and the relative contribution of the independent factors to the total variance was evaluated based on the “rdacca.hp” package [29]. To assess the relative importance of the deterministic and stochastic processes in the bacterial community assembly, the “picante” package was used to calculate the beta nearest taxon index (βNTI) and the Bray–Curtis-based Raup–Crick index (RC_bray_) [30]. In addition, linear regression analysis was performed using the “ggplot2” package to reveal the impact of the driving factors on the diversity and the βNTI of the bacterial community. The bacterial co-occurrence network was constructed based on a Spearman correlation matrix using the “ggClusterNet” package [31], and Gephi (v. 0.92) was used to visualize networks (|ρ| > 0.7 and *p* < 0.05) and calculate the global topological properties. Network vulnerability was calculated by the R script provided by a prior work [32]. The correlation between the network modules and the four organic acids was determined by the Mantel test using the “vegan” package. PICRUSt2 was used to reveal the functional composition of the bacterial communities [33].

## 3. Results and Discussion

### 3.1. Effects of Daqu Attributes on Core Microbes of APM

**Figure 1:**
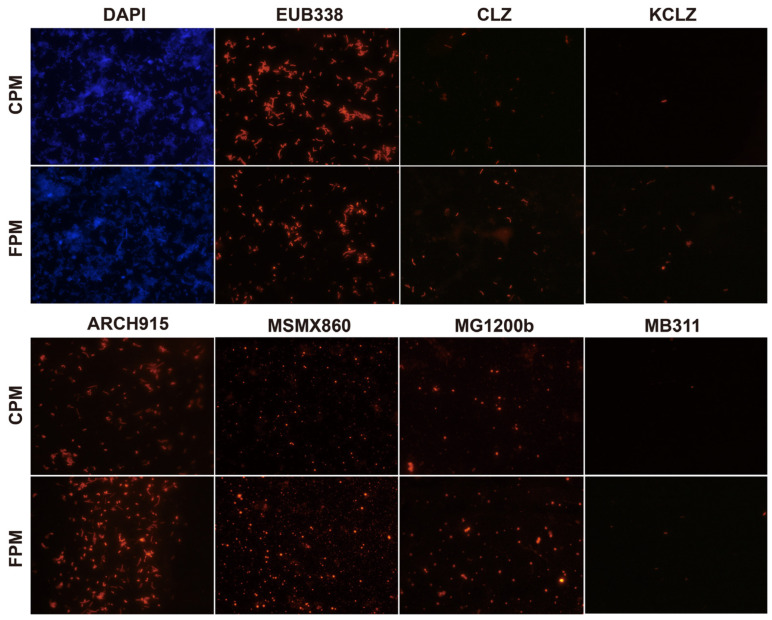
Differences in core microbes of fortified artificial pit mud (FPM) and conventional artificial pit mud (CPM) based on fluorescence in situ hybridization. DAPI, total microbes; EUB338, eubacteria; CLZ, *Clostridium*; KCLZ, *Clostridium kluyveri*; ARCH915, archaea; MSMX860, *Methanosarcinales*; MG1200b, *Methanomicrobiales*; MB311, *Methanobacteriales*.

The total number of microbes in the two APMs was similar, while the archaea number in the FPM was significantly higher than that in the CPM, and the eubacteria number was the opposite (Figure 1 and Table S2). The abundance and diversity of archaea can be used to evaluate the quality and age of the PM as few archaea usually inhabit the new PM [3,8,20]. Acetotrophic *Methanosarcinales* was the predominant archaea in both the APMs and was more abundant in FPM. *Methanosarcina* has been considered the vital indicator of pit age and has a high potential for flavor production, including hexanoic acid and ethyl hexanoate [34,35]. Moreover, hydrogenotrophic methanogen (*Methanomicrobiales* and *Methanobacteriales*) and the functional bacteria (*Clostridium* and *C. kluyveri*) in the FPM were also higher than that in the CPM (Figure 1 and Table S2). The interspecies hydrogen transfer between them contributed to the relieving of the inhibition effect of the hydrogen partial pressure on the latter, thereby promoting the formation of hexanoic acid and butyric acid [26]. These results indicated that the FD promoted the enrichment of core microbes in the APM.

### 3.2. Effects of Daqu Attributes on the Microbial Community of APM

A total of 79,418–124,416 and 84,529–113,345 effective reads were sequenced for the bacteria and fungi from all the samples, and the average number and ratio of high-quality sequences were 113,471 and 82.16%, and 86,050 and 85.12% (Table S3). The rarefaction curve of each sample demonstrated that the sequencing data were enough for subsequent analysis (Figure S1A,B). The results of the α-diversity showed that the richness (Chao1) and diversity (Shannon) of the bacteria and the diversity of the fungi in the FPM were significantly higher than in the CPM (Figure S1C,D). The Shannon index of the FPM ranged from 4.5 to 8.9 and was similar to the aged and high-quality PMs, suggesting a greater metabolic activity [25]. In addition, the explained rate of PCoA for the bacterial community (62.0%) was higher than that for the fungal community (39.4%), and the PREMANOVA test also showed that the bacterial β-diversity differed significantly (R^2^ = 0.438, *p* = 0.004) among the two kinds of APMs, while the fungal β-diversity was similar (R^2^ = 0.118, *p* = 0.241) (Figure S1E,F).

**Figure 2:**
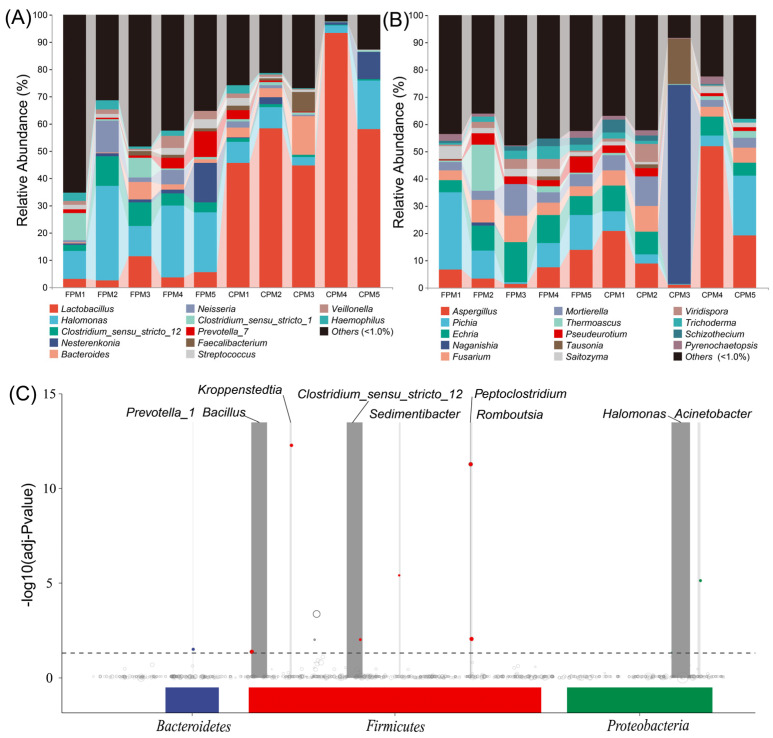
The bacterial (**A**) and fungal (**B**) communities of FPM and CPM at the genus level. The MetagenomeSeq analysis of bacterial community at the amplicon sequence variant (ASV) level, each node represents an ASV (**C**).

The bacterial communities of the two kinds of APMs were composed of 37 phyla, in which Firmicutes and Proteobacteria accounted for 69.49% to 98.47% of the total relative abundance (RA) (Figure S2A). The RA of Firmicutes in the CPM was remarkably higher than that in the FPM, while that of the Proteobacteria was the opposite. A total of 823 taxa were identified at the genus level, and 12 genera with an average RA >1.0% were defined as dominant (Figure 2A). Among them, a significantly higher abundance of *Lactobacillus* was observed in the CPM, while *Halomonas* and *Clostridium_sensu_stricto*_12 were remarkably enriched in the FPM (*t*-test, *p* < 0.05). The co-existence of *Clostridium_sensu_stricto*_12 and *Lactobacillus* has been observed in PM with different pit age [20,36], while *Halomonas* was not detected, and its contribution to SFB fermentation needs further study. For the fungi, 168 genera belonged to nine phyla, and Ascomycota was dominant in almost all the samples except CPM3, which was featured by Basidiomycota (Figure S2B). Correspondingly, CPM3 was governed by *Naganishia* at the genus level, while the other samples were represented by *Aspergillus*, *Pichia*, and *Echria* (Figure 2B). *Aspergillus* and *Pichia* were prevalent in the PM [15,17], whereas *Naganishia* and *Echria* have never been reported as the dominant genera, suggesting that PM may be a seed bank containing a large number of unknown microorganisms [34]. Additionally, there was also no significant difference in these dominant fungi in the two kinds of APMs (*t*-test, *p* > 0.05). In fact, the limited oxygen environment of PM was not suitable for the propagation of most fungi, resulting in a lower proportion and biomass [34,35]. However, the enormous fungal richness in *Daqu* may migrate into the PM during fermentation and accumulate gradually in the prolonged production process periodically [15]. Therefore, the fungal communities in PM might be mainly correlated with the pit age [20].

MetagenomeSeq analysis was performed to further explore the differences in the bacterial communities among the APMs. The results showed that the ASVs involving nine genera in the FPM were significantly up-regulated compared with the CPM (Figure 2C). *Bacillus* was the most remarkably increased genus except for *Clostridium_sensu_stricto*_12 and *Halomonas*, which could be attributed to the use of FD [27]. It has been reported that *Bacillus* was a contributor to various flavor compounds, such as hexanoic acid in the PM, and its RA was increased with the pit age significantly [9,22]. *Kroppenstedtia* might also originate from *Daqu* and could adapt to the anaerobic environment of the APM [9,37]. *Sedimentibacter* and *Prevotella* were common functional bacteria responsible for converting the macromolecular substance into organic acids in the PM; in particular, the former was positively correlated with pit age [25,38]. These results indicated that FD drives the directional evolution of the bacterial community, which may subsequently affect the material metabolism of the APM.

### 3.3. Effects of Daqu Attributes on Metabolic Profile of APM

**Figure 3:**
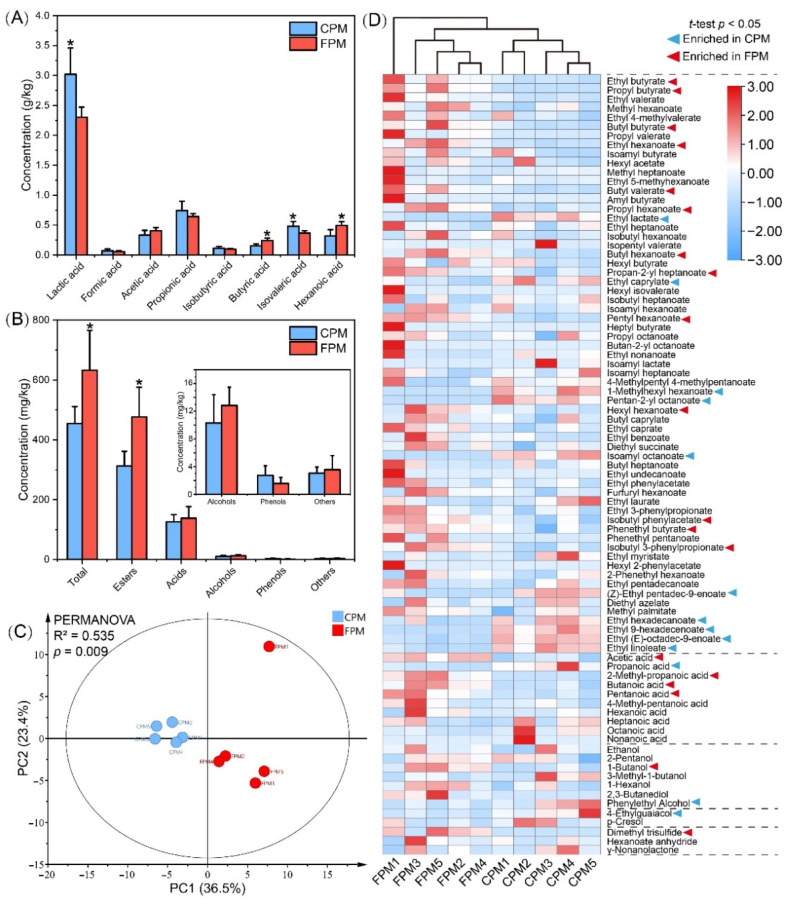
The concentration of major organic acids (**A**) and volatile compounds (**B**) in FPM and CPM, and the principal component analysis (**C**) and cluster heatmap analysis (**D**) of volatile compounds. * represents significant difference (*t*-test, *p* < 0.05).

The differences in the major organic acids of the APM are shown in Figure 3A; the content of lactic acid and isovaleric acid in the CPM was significantly higher than that in the FPM, whereas that of the hexanoic acid and butyric acid was the opposite. Consistent with the previous reports, lactic acid was the most abundant organic acid, and its content was negatively correlated with the PM quality and pit age [8,25]. Conversely, hexanoic acid and butyric acid were the feature metabolites of the PM consortia and usually increased with pit age [4,35]. In addition, the acetic acid content was high in the FPM, which might explain the enrichment of *Methanosarcinales*.

A total of 85 volatile compounds were detected from these APMs, including esters, alcohols, acids, phenols, and others (Table S4). The concentrations of all the compounds, except the phenols, in the FPM were higher than those in the CPM, especially the esters (*p* < 0.05) (Figure 3B). The results of the PCA and PERMANOVA tests further suggested that the difference in volatile profile between the CPM and the FPM was significant (R^2^ = 0.535, *p* = 0.009), which primarily resulted in 32 compounds (Figure 3C,D). Similarly to a previous study [35], esters were the most abundant and important flavor compounds in the PM, ranging from 65.27% to 79.74% of the total content. Here, the contents of the 23 esters were significantly different between the two APMs. Among them, 13 esters, including ethyl hexanoate and ethyl butyrate, were higher in the FPM, while 10 esters, such as ethyl lactate, were abundant in the CPM (Figure 3D). In general, ethyl hexanoate, ethyl butyrate, ethyl lactate, and ethyl acetate are important aroma contributors of SFB, and the ratio of ethyl hexanoate and ethyl lactate significantly affects its quality and characteristics [2,36]. Ethyl hexanoate gave a fruity, floral, and sweet flavor to the fresh SFB, while the contribution of ethyl lactate showed the greenness and the mushroom [39,40]. However, an excessive amount of the latter in SFB will lead to an irritating odor, acerbity, and bitterness. Therefore, an important strategy to elevate the quality of SFB is the achieving of “ethyl hexanoate-increasing” and “ethyl lactate-decreasing” by the bioturbation effect [19]. In addition, hexyl hexanoate and ethyl butyrate enriched in the FPM were conducive to the fruitiness of Baijiu, whereas ethyl palmitate and ethyl linoleate enriched in the CPM contributed little to the aroma characteristics due to their high odor threshold [40].

Acids accounted for 17.91% to 31.98% of the total content and were dominated by hexanoate. Consistent with the results obtained from HPLC (Figure 3A), GC-MS also showed that the concentrations of hexanoic acid, butyric acid, acetic acid, and pentanoic acid were higher in the FPM, while that of propionic acid was higher in the CPM (Figure 3D). These acids have been identified as the aroma-activity compounds of the SFB and contribute to the coordination of flavor and taste [2]. Alcohols with a relatively higher odor threshold accounted for 1.37% to 4.62% of the total content and were dominated by ethanol. Phenethyl alcohol and 1-butanol were enriched in the CPM and the FPM, respectively (Figure 3D), and the latter had the highest OAV among the alcohols in the SFB [40]. Moreover, 4-ethylguaiacol was more abundant in the CPM, while dimethyl trisulfide was only detected in the FPM. These results indicated that the flavor quality of the FPM was much better and uncovered the contribution of FD to the flavor characteristics of APM.

### 3.4. Correlation Analysis between Dominant Microbes and Metabolites

**Figure 4:**
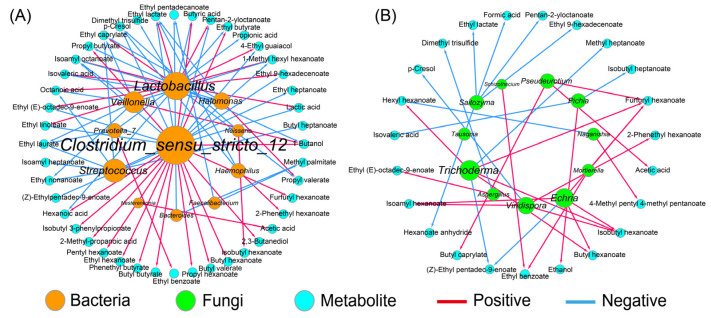
The correlation analysis between dominant microorganisms and metabolites in artificial pit mud. All links represent the significant correlation based on Spearman’s correlation coefficient (|ρ| > 0.7 and *p* < 0.05). The size of each microorganism node is proportional to its degree of connection with the volatiles; (**A**) bacteria; (**B**) fungi.

The correlation network between the dominant bacteria, as well as the fungi (RA > 1.0%), and the metabolites was constructed based on the Spearman method (Figure 4). The results showed that *Halomonas*, the predominant bacteria, was only positively correlated with propyl valerate, suggesting a weak ability for flavor producing. Conversely, *Clostridium_sensu_stricto*_12 with a relatively lower RA contributed greatly to the flavor quality of the APM, which promoted the formation of 16 compounds, such as hexanoic acid, butyric acid, and the corresponding ethyl esters (Figure 4A), which was consistent with prior works [20,36]. The flavor profile of the APM was also affected by *Lactobacillus*, which was positively associated with 11 compounds, such as lactic acid and ethyl lactate, but negatively with 7 compounds, such as hexanoic acid and ethyl butyrate (Figure 4A). This is different from the weak correlation between *Lactobacillus* and the volatile compounds observed in the aged PM, probably because of the decreased *Lactobacillus* and the increased flavor-producing microbes, such as *Caproiciproducens* and *Methanosarcina* [35].

By contrast, the contribution of the fungal community to the flavor quality was weak (Figure 4B), which was consistent with the previous study [41]. *Aspergillus* and *Pichia* were the common functional fungi in the PM and were related to the formation of esters and alcohols, respectively [13,14], which was supported by the present study (Figure 4B). However, there are no reports on the metabolic function of *Echria* and *Naganishia* in Baijiu fermentation. Here, the former showed a stronger metabolic activity as it was positively associated with butyl hexanoate, isobutyl hexanoate, isopentyl hexanoate, and furfuryl hexanoate, while the latter was negatively correlated with hexyl hexanoate (Figure 4B). In addition, *Trichoderma* also promoted the production of four esters, which might be attributed to its extensive enzyme activities, including cellulase, xylanase, and chitinase [42].

### 3.5. Driving Factors for Variation and Assembly of Bacterial Community

The differences in physicochemical properties between the two kinds of APMs are shown in Figure S3. The FPM had a significantly higher pH and NH_4_^+^-N content and a lower TA content compared with the CPM (*p* < 0.05), while the contents of AP, TE, and moisture were similar. It was reported that pH and TA were the important properties to evaluate the quality and age of PM, and high-quality or aged PM usually had a natural pH and a low TA [25,35]. The NH_4_^+^-N content was also positively correlated with pit age and played an important role in the growth and reproduction of the PM microbial consortia [8,22].

**Figure 5:**
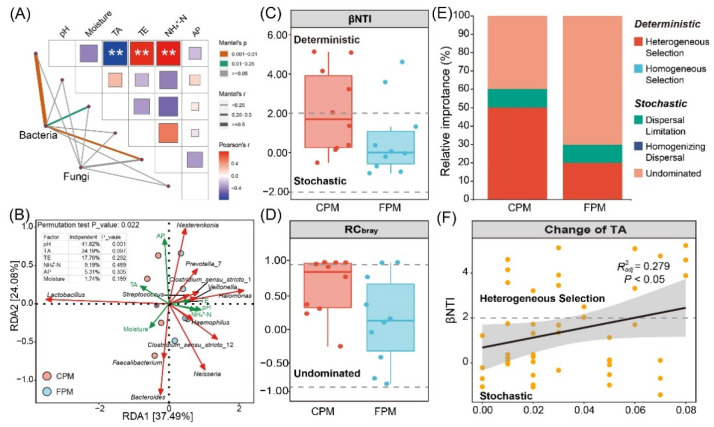
Determination of the driving factor for variation and assembly of bacterial community in artificial pit mud. (**A**) Mantel test of overall communities and physicochemical factors. (**B**) Redundancy analysis and hierarchical partitioning analysis of dominant bacterial genera and physicochemical factors. (**C**) βNTI results of the bacterial community. (**D**) RC_bray_ results of the bacterial community. (**E**) Relative importance of deterministic and stochastic assembly processes in shaping bacterial community. (**F**) Linear regression analysis between βNTI and change of TA.

Mantel tests between the overall communities and the physicochemical properties were conducted to determine the driving factors of the community variation (Figure 5A). The results suggested that pH, TA, and NH_4_^+^-N significantly influenced the bacterial community but not the fungal community. In addition, the α- and β-diversity of the bacterial communities were also positively correlated with pH and NH_4_^+^-N but negatively with TA (Figure S4). RDA was used to further reveal the correlation between the dominant bacterial genera and the physicochemical properties (Figure 5B). A high explanation rate (61.57%) of the total variation and a low *p*-value (0.022) of the whole-model permutation test demonstrated the reliability of the RDA model. *Lactobacillus* was positively associated with TA, and most of the other genera, such as *Clostridium_sensu_stricto*_12, were positively correlated with pH and NH_4_^+^-N. Notably, the hierarchical partitioning analysis showed that 41.82% of the total variation can be independently explained by pH (*p* = 0.001), which was consistent with Tao et al. [8], who reported that pH was the major variable for explaining the variance in the prokaryotic community structure, indicating the pivotal role of pH in regulating the bacterial community in APM.

To explore the effect of FD on the bacterial community assembly of APM, βNTI and RC_bray_ were calculated to quantify the determinism and stochasticity [30]. The results of βNTI showed that both the determinism (|βNTI| > 2) and the stochasticity processes (−2 < βNTI < 2) governed the community assembly of the CPM, whereas the latter controlled that of the FPM (Figure 5C). Furthermore, the distribution of RC_bray_ values suggested that undominated (|RC_bray_| < 0.95) was the primary stochasticity process in the two kinds of APMs (Figure 5D), accounting for 40% and 60% of the CPM and FPM, respectively (Figure 5E). Both the stochastic and the deterministic processes will affect the community assembly of the ecosystem, but the former was more important as the availability of resources increased [43], which was in line with the NH_4_^+^-N content of the two APMs (Figure S3). Similarly, the stochastic process was more dominant for the bacterial community assembly in soil aggregates with a higher soil organic carbon and total nitrogen level [44]. In addition, the increase in the stochastic process in the FPM may imply that adapted lineages of bacteria accumulate, resulting in weaker niche-based exclusion and higher biodiversity [43,45], which explains the significantly higher α-diversity in the FPM (Figure S1). Conversely, the contribution of heterogeneous selection for the community assembly in the CPM was higher than in the FPM (Figure 5E). According to the previous study, heterogeneous selection usually occurs when the changing of environmental pressures across space or time leads to a narrower niche breadth [46]. For example, only species that can resist high acid stress and oligotrophic conditions can survive in the surface water of an acid mine drainage lake due to the strong heterogeneous selection [47]. Notably, our results also indicated that the relative importance of heterogeneous selection increased with the TA content (Figure 5F). Therefore, the high proportion of heterogeneous selection in CPM might be closely related to its extreme environmental conditions, especially the high TA content, which inhibited the growth and metabolism of most acid-intolerant microorganisms, such as *Clostridium* and methanogen [48].

### 3.6. Co-Occurrence Network and Metabolic Functional of Bacterial Community

**Figure 6:**
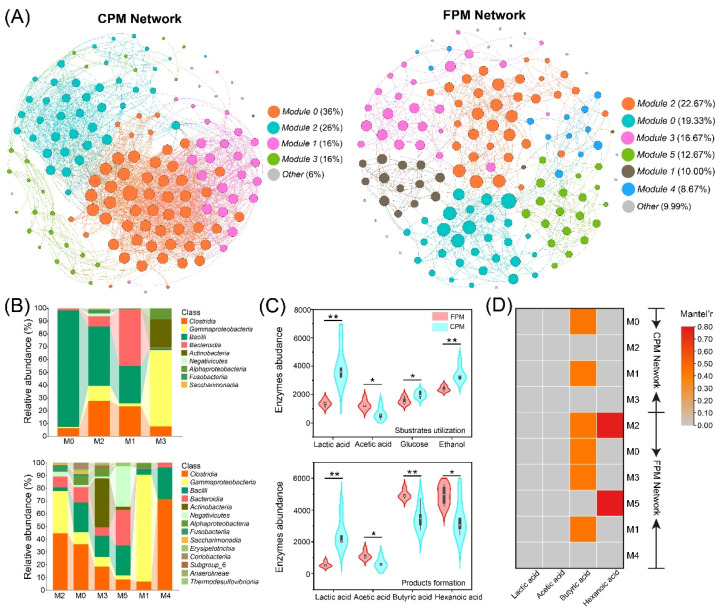
Bacterial co-occurrence networks and the community composition and potential function of primary modules. (**A**) The co-occurrence network analysis of bacterial community ASVs: each dot represents a bacterial ASV, and the links represent statistical significance (|ρ| > 0.7, *p* < 0.05). (**B**) The community composition of primary modules at the class level. (**C**) The abundance variation of functional genes encoding for key enzymes involved in substrate utilization and product formation in bacterial communities of FPM and CPM. Significant values based on *t*-test are shown as: * *p* < 0.05; ** *p* < 0.01. (**D**) Mantel test between primary modules and important organic acids; correlations with *p* > 0.01 are marked in grey.

Co-occurrence networks for CPM and FPM were constructed based on the ASV level to reveal the effect of FD on the interaction of the bacterial community. As shown in Figure 6A, the CPM and FPM networks differed significantly and contained four and six modules, respectively. The global topological properties indicated that the CPM network showed higher complexity according to its edge number, average degree, and density, while the FPM network became less vulnerable and more modular, as measured by the vulnerability and modularity values (Table 1). In addition, the proportion of negative edges in the FPM network was significantly higher than that in the CPM network, suggesting that the former had stronger robustness [32,49]. The microbial composition of the main modules was also remarkably different between the two networks (Figure 6B). M0 and M2 in the CPM network were dominated by *Bacilli*, whereas M2, M0, and M4 in the FPM network were governed by *Clostridia*. Meanwhile, *Bacteroidia* was also prevalent in all the modules (except M1) of the FPM network. According to Hu et al. [25], some hub microorganisms in normal or high-quality PMs were affiliated with *Clostridia* and *Bacteroidia*, while the RA of *Bacilli* was significantly higher than those of other taxa in degraded PMs. Therefore, the enhanced stability of the FPM network may be caused by the co-occurrence of *Clostridia* and *Bacteroidia* with high abundance in the main modules.

The primary biological function of the PM microbial consortia in the SFB brewing micro-ecosystem is to synthesize hexanoic acid and butyric acid from lactic acid, ethanol, and other electron donors through the chain elongation process [4,26,50]. Therefore, the abundances of enzymes related to substrate utilization and product formation were predicted based on PICRUSt2 (Table S5). The results showed that the CPM could better utilize lactic acid, glucose, and ethanol, while FPM had higher potential in the synthesis of hexanoic acid, butyric acid, and acetic acid (Figure 6C), which was consistent with the contents of the corresponding organic acids (Figure 3A). In general, the PM microbiota preferred to metabolize lactic acid to hexanoic acid [35,51], but the lactic acid-driven hexanoic acid production requires a special consideration of the product selectivity. For instance, (i) different community compositions favor disparate fermentation pathways and yield various acid production efficiencies [52]; (ii) the efficiency of hexanoic acid synthesis will be negatively affected by the lower pH because most hexanoic acid-producing bacteria exhibit greater yields under neutral conditions [53]; (iii) lactic acid can also be converted to propionic acid, which will cause carbon diversion from hexanoic acid to produce propionic acid [54]. In the present study, abundant *Clostridium* and methanogens were observed in the FPM, while lower pH and higher propionic acid content were detected in the CPM, which might be the reason for the higher content of hexanoic acid and butyric acid in the former. Moreover, the Mantel test showed that all the modules (except M4) in the FPM network were significantly correlated with the hexanoic acid and/or butyric acid content, while only M0 and M1 in the CPM network were remarkably associated with the butyric acid content (Figure 6D), which further explained the higher content of hexanoic acid and butyric acid in the FPM. In particular, the correlations between M2 and M5 of the FPM network and hexanoic acid content might be related to a higher RA of *Negativicutes*, because some members of *Negativicutes* can also convert lactic acid into hexanoic acid [53].

## 4. Conclusions

This study provided a comprehensive insight into the importance of *Daqu* attributes on the APM quality by polyphasic detection methods. The results showed that FD promoted the directional evolution of the prokaryotic community by changing the physicochemical properties of the APM, especially the pH and TA content. *Clostridium_sensu_stricto*_12 exhibited greater potential in the formation of flavor substances and increased its abundance in the PM, which may represent a way to achieve “ethyl hexanoate-increasing” and “ethyl lactate-decreasing”. Furthermore, FD induced a co-occurrence of *Clostridia* and *Bacteroidia* with a high abundance and, thereby, enhanced the community stability and metabolic function. Our findings may contribute to the optimizing of the APM manufacturing technology and to the further production of high-quality SFB.

## Figures and Tables

**Table 1:** The global topological properties of co-occurrence networks.

Network Properties ^1^	FPM	CPM
Node number	150	150
Edge number	602	1493
Proportion of positive edge	64.12%	91.06%
Proportion of negative edge	35.88%	8.91%
Average degree	8.027	19.907
Average path length	3.553	2.945
Average clustering coefficient	0.236	0.135
Diameter	8.164	8.152
Density	0.054	0.134
Modularity	0.626	0.340
Modules number	21	13
Vulnerability	0.011	0.009

^1^ All properties were measured using the interactive platform Gephi, except vulnerability, which was calculated by the R code provided by a prior work [32].

## Data Availability

All raw sequences were deposited in the NCBI Sequence Read Archive under accession number PRJNA835955.

## Supplementary Materials

The following supporting information can be downloaded at: https://www.mdpi.com/article/10.3390/foods11182922/s1, Figure S1: differences in rarefaction curves (A, bacteria; B, fungi), α-diversity (C, bacteria; D, fungi) and β-diversity (E, bacteria; F, fungi) of microbial community between FPM and CPM, Figure S2: the composition of microbial community at the phylum level. (A) bacteria; (B) fungi, Figure S3: variations in physicochemical parameters between FPM and CPM. Significant values based on *t*-test are shown as: * *p* < 0.05, ** *p* < 0.01, Figure S4: linear regression analysis between potential driving factors and α/β-diversity of bacterial community, Table S1: the detailed information of fluorescently labeled oligo-probes, Table S2: differences in core microbes of FPM and CPM based on fluorescence in situ hybridization (×10^7^ cells/g), Table S3: sequencing results of all artificial pit mud samples, Table S4: the detail of volatile compounds detected in CPM and FPM, Table S5: the abundance of functional genes encoding for key enzymes involved in substrate utilization and product formation in bacterial communities.
