# Supplementary material for: Effects of Daqu Attributes on Distribution and Assembly Patterns of Microbial Communities and Their Metabolic Function of Artificial Pit Mud
Source: Foods. 2022 Sep 19;11(18):2922. doi: 10.3390/foods11182922 (PMC9498905; doi:10.3390/foods11182922)
Supplement: Supplementary file 1 [file foods-11-02922-s001.zip › foods-1895431-supplementary.pdf]

Supplementary materials

Effects of *Daqu* attributes on distribution and assembly patterns of microbial communities and their metabolic function of artificial pit mud

Yu Mu[1], Jun Huang[1], Rongqing Zhou[1,2,*], Suyi Zhang[2,3], Hui Qin[2,3], Hanlan Tang[2,3], Qianglin Pan[1], Huifang Tang[1]

[1] College of Biomass Science and Engineering, Sichuan University, Chengdu 610065, China;

[2] National Engineering Research Center of Solid-State Manufacturing, Luzhou 646000, China;

[3] Luzhou Lao Jiao Co., Ltd., Luzhou 646699, China.

*** The complete contact details of the corresponding author:**

Name: Rongqing Zhou;

E-mail: zhourqing@scu.edu.cn;

Phone: +86-28-85406149;

Fax: +86-28-85405237;

Mailing address: College of Biomass Science and Engineering, Sichuan University, Chengdu 610065, China.

Table S1 The detailed information of fluorescently labeled oligo-probes.

Probe/Stain	Sequence (5′-3′)	Target
DAPI	—	Total microbes
EUB338	GCTGCCTCCCGTAGGAGT	Eubacteria
CLZ	GGCTACCTTGTTACGACTTCACCCCA	*Clostridium*
KCLZ	CCTGCACACCCTTTACGCCCAGTAATTCCGGACAA	*Clostridium kluyveri*
ARCH915	GTGCTCCCCCGCCAATTCCT	Archaea
MSMX860	GGCTCGCTTCACGGCTTCCCT	*Methanosarcinales*
MG1200b	CRGATAATTCGGGGCATGCTG	*Methanomicrobiales*
MB311	ACCTTGTCTCAGGTTCCATCTCC	*Methanobacteriales*

Table S2 Differences in core microbes of FPM and CPM based on fluorescence in situ hybridization ($\times 10^7$ cells/g).

Probe/Stain	FPM	CPM
DAPI	8724.00±524.25	8045.47±717.24
EUB338	1393.90±715.00b	3354.70±514.42a
CLZ	152.67±43.32a	67.85±11.11b
KCLZ	34.90±11.95a	10.91±7.27b
ARCH915	4232.11±658.86a	1380.82±287.42b
MSMX860	2060.80±557.44a	455.10±109.51b
MG1200b	364.95±144.71a	113.28±14.06b
MB311	12.12±4.20a	0.00±0.00b

Note: Different letters in the same row indicate significant differences in *t*-test ($p < 0.05$).

Table S3 Sequencing results of all artificial pit mud samples.

Sample	Bacteria			Fungi		
	Effective sequences [a]	High-quality sequences [b]	Ratio (%) [c]	Effective sequences [a]	High-quality sequences [b]	Ratio (%) [c]
FPM1	107283	86984	81.08	118560	115921	81.22
FPM2	108118	92573	85.62	124416	120940	83.56
FPM3	101193	72564	71.71	116659	112408	82.33
FPM4	113345	98689	87.07	117954	116365	83.08
FPM5	111563	96213	86.24	119358	118731	86.15
CPM1	98198	82040	83.55	122058	120745	84.39
CPM2	95112	83755	88.06	124318	122058	82.81
CPM3	84529	65672	77.69	79418	75653	69.62
CPM4	92895	90906	97.86	122233	121424	86.75
CPM5	98683	91109	92.32	115368	110471	81.65

[a] The effective sequences were obtained by removing low-quality tags.

[b] The high-quality sequences were obtained by further removing chimeras.

[c] The ratio of high-quality sequences in effective sequences

Table S4 The detail of volatile compounds detected in CPM and FPM.

Compounds name	No. CAS	Concentration (mg/kg)										*t*-test
		CPM1	CPM2	CPM3	CPM4	CPM5	FPM1	FPM2	FPM3	FPM4	FPM5	*p*_value
**Esters**												
Ethyl butyrate	105-54-4	0.90	0.34	0.61	0.56	0.23	3.69	1.33	1.22	1.33	2.50	0.034
Propyl butyrate	105-66-8	0.00	0.00	0.00	0.00	0.00	0.12	0.06	0.06	0.06	0.14	0.007
Ethyl valerate	539-82-2	2.51	0.97	0.99	1.63	0.80	9.52	2.65	3.48	2.44	3.83	—
Methyl hexanoate	106-70-7	0.86	0.73	1.28	1.49	0.85	1.49	1.90	1.11	1.23	2.09	—
Ethyl 4-methylvalerate	25415-67-2	0.14	0.06	0.08	0.06	0.03	0.17	0.08	0.08	0.06	0.14	—
Butyl butyrate	109-21-7	0.60	0.17	0.07	0.20	0.05	1.24	1.09	0.99	1.05	2.61	0.006
Propyl valerate	141-06-0	0.00	0.00	0.00	0.00	0.00	0.24	0.06	0.02	0.06	0.00	—
Ethyl hexanoate	123-66-0	110.71	106.83	91.89	103.00	100.73	130.23	153.11	178.26	125.12	209.21	0.019
Isoamyl butyrate	106-27-4	0.36	0.21	0.11	0.09	0.09	0.30	0.15	0.19	0.19	0.38	—
Hexyl acetate	142-92-7	0.07	0.21	0.08	0.07	0.05	0.16	0.13	0.08	0.08	0.16	—
Methyl heptanoate	106-73-0	0.04	0.09	0.08	0.11	0.09	0.47	0.09	0.05	0.05	0.09	—
Ethyl 5-methyhexanoate	10236-10-9	0.10	0.07	0.05	0.06	0.07	0.25	0.05	0.06	0.03	0.07	—
Butyl valerate	591-68-4	0.78	0.21	0.07	0.41	0.14	3.15	1.22	1.43	1.08	2.37	0.015
Amyl butyrate	540-18-1	0.00	0.00	0.00	0.00	0.00	0.80	0.00	0.00	0.00	0.00	—
Propyl hexanoate	626-77-7	0.00	3.05	0.17	6.51	4.81	6.07	9.64	16.52	9.57	19.00	0.009
Ethyl lactate	687-47-8	9.64	6.21	5.65	8.16	4.81	1.83	0.00	0.00	0.00	0.25	0.001
Ethyl heptanoate	106-30-9	19.36	17.80	6.76	15.09	19.76	32.74	12.11	15.52	8.53	16.62	—
Isobutyl hexanoate	105-79-3	2.27	0.92	0.61	0.90	1.22	0.83	1.15	1.94	1.70	3.20	—
Isopentyl valerate	2050-09-1	0.30	0.16	3.84	0.15	0.15	0.48	0.18	0.26	0.17	0.34	—
Butyl hexanoate	626-82-4	49.31	13.93	7.51	24.03	13.14	40.42	73.58	104.36	78.39	135.97	0.006
Hexyl butyrate	2639-63-6	1.61	1.59	0.67	0.90	0.69	2.09	1.82	1.25	1.18	0.39	—

Propan-2-yl heptanoate	34997-46-1	0.45	0.23	0.06	0.37	0.24	0.84	0.33	0.68	0.35	0.59	0.041
Ethyl caprylate	106-32-1	28.91	40.62	13.69	27.77	33.80	25.89	10.88	15.45	9.12	17.40	0.038
Hexyl isovalerate	10032-13-0	0.00	0.00	0.00	0.00	0.00	0.17	0.00	0.00	0.00	0.00	—
Isobutyl heptanoate	7779-80-8	0.07	0.05	0.03	0.05	0.09	0.13	0.00	0.00	0.00	0.07	—
Isoamyl hexanoate	2198-61-0	17.82	10.20	9.46	11.35	14.77	7.01	10.02	18.44	12.27	18.70	—
Pentyl hexanoate	540-07-8	12.40	8.15	3.81	10.32	8.34	28.43	23.04	30.51	17.20	26.70	0.001
Heptyl butyrate	5870-93-9	0.00	0.00	0.00	0.00	0.00	0.43	0.00	0.00	0.00	0.00	—
Propyl octanoate	624-13-5	0.51	0.48	0.00	0.79	0.48	0.63	0.28	0.69	0.30	0.55	—
Butan-2-yl octanoate	5458-61-7	0.00	0.00	0.00	0.00	0.00	0.18	0.00	0.00	0.00	0.00	—
Ethyl nonanoate	123-29-5	0.16	0.36	0.15	0.18	0.29	0.79	0.07	0.11	0.05	0.08	—
Isoamyl lactate	19329-89-6	0.00	0.00	0.24	0.00	0.06	0.00	0.00	0.00	0.00	0.00	—
Isoamyl heptanoate	109-25-1	0.51	0.52	0.28	0.53	0.91	0.82	0.20	0.60	0.25	0.39	—
4-Methylpentyl 4-methylpentanoate	35852-42-7	0.21	0.09	0.12	0.15	0.16	0.31	0.00	0.00	0.12	0.00	—
1-Methylhexyl hexanoate	6624-58-4	0.66	0.39	0.16	0.83	0.59	0.00	0.00	0.00	0.00	0.00	0.010
Pentan-2-yl octanoate	55193-30-1	0.21	0.13	0.11	0.16	0.08	0.00	0.00	0.00	0.00	0.00	0.003
Hexyl hexanoate	6378-65-0	52.14	59.00	33.98	51.07	42.39	48.94	80.13	116.08	65.30	83.95	0.031
Butyl caprylate	589-75-3	1.79	1.16	0.34	2.40	1.30	1.37	1.31	2.63	1.57	2.41	—
Ethyl caprate	110-38-3	2.10	1.28	1.99	2.82	2.04	4.41	3.14	3.33	2.00	2.50	—
Ethyl benzoate	93-89-0	0.23	0.33	0.03	0.04	0.02	0.16	0.14	0.99	0.29	0.46	—
Diethyl succinate	123-25-1	0.19	0.11	0.20	0.24	0.20	0.21	0.19	0.30	0.24	0.28	—
Isoamyl octanoate	2035-99-6	0.62	0.71	0.39	0.80	0.89	0.00	0.00	0.00	0.00	0.00	0.001
Butyl heptanoate	5454-28-4	2.71	5.87	1.77	3.88	4.68	9.61	3.15	5.83	2.44	2.92	—
Ethyl undecanoate	627-90-7	0.00	0.00	0.00	0.00	0.00	0.22	0.00	0.00	0.00	0.00	—
Ethyl phenylacetate	101-97-3	3.66	2.45	3.11	3.85	2.16	6.36	3.22	5.43	2.20	2.88	—
Furfuryl hexanoate	39252-02-3	0.35	0.23	0.00	0.28	0.19	0.14	0.33	0.73	0.35	0.57	—
Ethyl laurate	106-33-2	0.62	0.57	0.96	1.45	1.85	1.18	0.52	0.80	0.32	0.43	—

Ethyl 3-phenylpropionate	2021-28-5	8.37	5.06	8.17	9.15	5.72	12.07	8.16	11.50	6.51	8.85	—
Isobutyl phenylacetate	102-13-6	0.40	0.25	0.00	0.48	0.00	0.77	0.63	0.79	0.45	0.51	0.010
Phenethyl butyrate	103-52-6	0.33	0.24	0.00	0.34	0.18	0.38	0.34	0.45	0.36	0.41	0.031
Phenethyl pentanoate	7460-74-4	0.00	0.00	0.00	0.00	0.00	0.45	0.00	0.00	0.00	0.34	—
Isobutyl 3-phenylpropionate	28048-94-4	0.67	0.29	0.16	0.77	0.36	0.86	1.07	1.69	1.01	1.29	0.004
Ethyl myristate	124-06-1	0.92	0.58	1.43	1.98	1.14	0.76	0.77	1.25	0.94	0.89	—
Hexyl 2-phenylacetate	5421-17-0	0.00	0.00	0.00	0.00	0.00	0.36	0.00	0.00	0.00	0.00	—
2-Phenethyl hexanoate	6290-37-5	3.72	2.48	1.95	4.26	4.86	1.80	3.80	7.52	4.45	4.98	—
Ethyl pentadecanoate	41114-00-5	0.27	0.22	0.40	0.49	0.33	0.52	0.30	0.57	0.33	0.28	—
(Z)-Ethyl pentadec-9-enoate	56219-09-1	0.00	0.08	0.11	0.11	0.09	0.07	0.00	0.00	0.00	0.00	0.032
Diethyl azelate	624-17-9	0.13	0.10	0.19	0.19	0.12	0.08	0.09	0.22	0.14	0.14	—
Methyl palmitate	112-39-0	0.00	0.10	0.16	0.17	0.10	0.23	0.16	0.18	0.12	0.15	—
Ethyl hexadecanoate	628-97-7	15.35	11.22	16.34	16.73	14.49	11.62	10.54	15.25	10.76	9.91	0.047
Ethyl 9-hexadecenoate	54546-22-4	1.19	0.76	1.07	1.63	1.01	0.00	0.00	0.00	0.00	0.00	0.001
Ethyl (E)-octadec-9-enoate	6114-18-7	6.59	5.06	6.64	6.30	5.98	3.16	2.84	2.93	2.47	1.96	0
Ethyl linoleate	7619-8-1	6.92	5.22	8.60	6.97	6.81	3.82	2.45	2.68	2.17	1.97	0
**Acids**												
Acetic acid	64-19-7	1.25	1.67	1.83	1.93	1.23	2.54	2.73	2.76	2.71	2.24	0.009
Propanoic acid	79-09-4	0.89	0.97	1.12	2.31	0.76	0.54	0.18	0.30	0.19	0.36	0.009
2-Methyl-propanoic acid	74381-40-1	0.32	0.29	0.29	0.34	0.16	0.71	0.52	1.02	0.63	1.08	0.008
Butanoic acid	107-92-6	7.28	7.94	8.01	11.95	4.43	13.11	15.52	22.68	15.17	21.46	0.003
Pentanoic acid	109-52-4	4.67	5.28	3.53	6.57	3.49	13.69	8.88	15.15	6.70	8.68	0.018
4-Methyl-pentanoic acid	646-07-1	0.19	0.20	0.24	0.19	0.14	0.26	0.18	0.38	0.17	0.24	—
Hexanoic acid	142-62-1	66.21	65.36	63.87	80.34	67.00	50.99	84.95	124.27	66.16	79.10	—
Heptanoic acid	111-14-8	10.62	24.64	10.65	18.71	20.76	19.37	11.63	22.19	9.24	10.82	—
Octanoic acid	124-07-2	12.31	48.66	12.25	23.73	24.48	12.71	7.64	15.53	6.83	8.95	—

Nonanoic acid	112-05-0	0.00	0.89	0.00	0.00	0.00	0.00	0.00	0.00	0.00	0.00	—
**Alcohols**												
Ethanol	64-17-5	4.95	4.27	13.18	8.26	7.12	7.91	7.27	12.43	8.50	7.38	—
2-Pentanol	6032-29-7	0.08	0.07	0.07	0.04	0.00	0.03	0.07	0.07	0.06	0.07	—
1-Butanol	71-36-3	0.18	0.13	0.31	0.83	0.00	0.50	1.09	1.73	1.20	1.83	0.008
3-Methyl-1-butanol	123-51-3	0.18	0.17	0.39	0.26	0.30	0.16	0.16	0.23	0.20	0.23	—
1-Hexanol	111-27-3	0.96	1.61	1.93	1.23	1.05	1.50	2.13	2.13	1.50	2.22	—
2,3-Butanediol	19132-06-0	0.00	0.00	0.00	0.00	0.00	0.30	0.00	0.24	0.10	0.71	—
Phenylethyl Alcohol	60-12-8	0.55	0.43	0.83	0.95	1.13	0.38	0.57	0.57	0.36	0.36	0.043
**Phenols**												
4-Ethylguaiacol	2785-89-9	0.25	0.20	0.30	0.29	0.58	0.14	0.17	0.20	0.11	0.13	0.034
p-Cresol	106-44-5	1.71	4.21	3.69	0.97	1.41	2.78	1.76	0.94	0.70	0.83	—
**Others**												
Dimethyl trisulfide	3658-80-8	0.00	0.00	0.00	0.00	0.00	0.05	0.05	0.00	0.04	0.08	0.009
Hexanoate anhydride	2051-49-2	3.58	2.17	1.98	3.90	2.72	1.20	2.88	6.63	2.51	3.46	—
γ-Nonanolactone	104-61-0	0.16	0.14	0.21	0.27	0.16	0.11	0.19	0.24	0.14	0.14	—

Note: "—" represents that there is no significant difference in the concentration of compounds between CPM and FPM (*t*-test, $p > 0.05$).

Table S5 The abundance of functional genes encoding for key enzymes involved in substrate utilization and product formation in bacterial communities.

Substrate utilization	Function description	Enzymes	Enzymatic abundance/(per million EC)									
			FPM1	FPM2	FPM3	FPM4	FPM5	CPM1	CPM2	CPM3	CPM4	CPM5
Lactic acid	L-lactate dehydrogenase (cytochrome)	EC:1.1.2.3	348.02	752.00	356.43	604.75	684.84	284.66	277.99	105.48	93.95	557.05
	D-lactate dehydrogenase (cytochrome)	EC:1.1.2.4	62.91	45.77	93.72	60.39	97.82	23.76	37.40	11.07	10.33	66.41
	Lactate 2-monooxygenase	EC:1.13.12.4	4.42	1.13	6.42	1.96	4.63	0.68	0.02	0.35	0.00	0.93
	L-lactate dehydrogenase	EC:1.1.1.27	668.96	567.66	1270.88	614.76	670.31	2888.81	3575.46	3129.23	6179.44	2964.50
Glucose	Hexokinase	EC:2.7.1.1	11.92	5.44	33.72	23.21	15.96	24.00	30.79	74.22	0.69	1.96
	Glucokinase	EC:2.7.1.2	1593.98	1260.15	1816.14	1353.59	1170.57	1817.82	1837.74	2187.93	2173.30	1491.91
	Polyphosphate--glucose phosphotransferase	EC:2.7.1.63	72.96	38.64	67.06	54.67	244.55	23.68	59.30	11.50	29.90	186.65
	ADP-specific phosphofructokinase	EC:2.7.1.146	0.55	0.89	4.85	3.25	2.35	3.62	1.86	10.82	0.00	0.00
Ethanol	Alcohol dehydrogenase	EC:1.1.1.1	2270.85	2524.85	2668.90	2274.71	2305.00	3046.61	3324.09	3095.68	4367.86	3330.12
	Alcohol dehydrogenase (NADP(+))	EC:1.1.1.2	0.08	0.67	0.88	0.63	0.05	0.36	0.09	0.84	0.00	0.00
Acetic acid	Acetate--CoA ligase	EC:6.2.1.1	1147.33	1257.30	917.11	1250.65	1785.85	557.44	631.20	394.91	213.84	1319.50
Product formation												
Lactic acid	D-lactate dehydrogenase	EC:1.1.1.28	554.20	488.76	818.77	470.25	442.92	1981.62	2398.23	2048.53	4105.54	1961.69
Acetic acid	Aldehyde dehydrogenase (NAD(P)(+))	EC:1.2.1.5	2.85	0.01	2.14	0.68	0.78	1.40	0.59	1.47	0.19	1.18
	Aldehyde dehydrogenase (NAD(+))	EC:1.2.1.3	1035.42	966.45	1160.23	994.02	1550.07	530.23	652.46	494.25	187.08	1115.48
	Acetate--CoA ligase (ADP-forming)	EC:6.2.1.13	6.24	0.07	2.25	0.63	0.03	2.06	0.67	2.33	0.00	0.39
Butyric acid	Acetyl-CoA C-acetyltransferase	EC:2.3.1.9	2371.16	2766.36	2240.43	2575.36	3167.91	1947.91	2367.49	1601.48	2453.47	3332.50
	3-hydroxybutyryl-CoA dehydrogenase	EC:1.1.1.157	1222.08	1482.82	1176.85	1320.38	1468.05	610.30	660.68	439.79	204.51	1126.62
	3-hydroxybutyryl-CoA dehydratase	EC:4.2.1.55	41.21	72.89	170.20	87.88	60.40	66.04	43.67	198.00	4.76	17.84
	Butyrate kinase	EC:2.7.2.7	401.65	229.26	426.94	258.16	298.22	327.74	231.69	323.01	25.48	80.97

	Acetate CoA-transferase	EC:2.8.3.8	448.84	251.72	486.12	247.82	152.96	256.50	232.37	235.45	27.18	79.84
	Phosphate butyryltransferase	EC:2.3.1.19	338.58	186.62	374.50	223.07	273.23	260.23	179.88	253.64	20.12	68.35
Hexanoic acid	Acetyl-CoA C-acetyltransferase	EC:2.3.1.9	2371.16	2766.36	2240.43	2575.36	3167.91	1947.91	2367.49	1601.48	2453.47	3332.50
	3-hydroxybutyryl-CoA dehydrogenase	EC:1.1.1.157	1222.08	1482.82	1176.85	1320.38	1468.05	610.30	660.68	439.79	204.51	1126.62
	3-hydroxybutyryl-CoA dehydratase	EC:4.2.1.55	41.21	72.89	170.20	87.88	60.40	66.04	43.67	198.00	4.76	17.84
	3-hydroxyacyl-CoA dehydrogenase	EC:1.1.1.35	769.73	1119.78	633.32	981.99	922.82	423.15	411.94	195.40	146.38	778.98
	Acyl-CoA hydrolase	EC:3.1.2.20	2.73	1.91	5.08	3.22	5.14	0.98	1.83	0.75	0.32	2.76

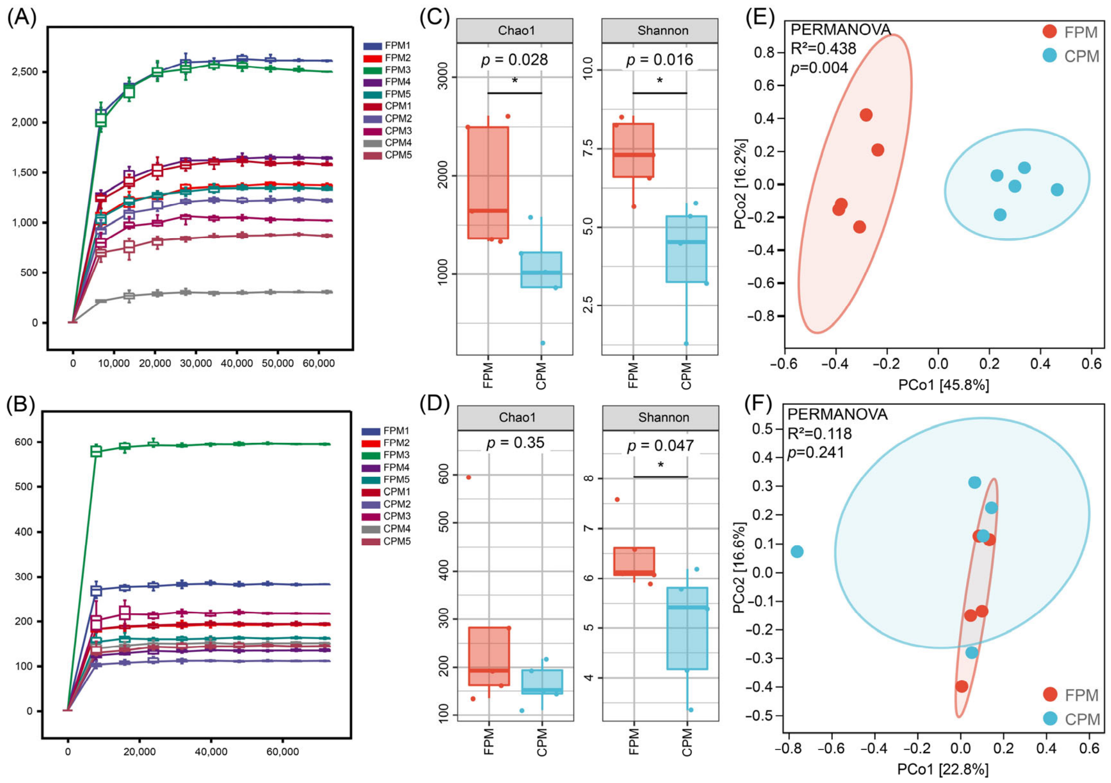

Figure S1 Differences in rarefaction curves (A, bacteria; B, fungi), α-diversity (C, bacteria; D, fungi) and β-diversity (E, bacteria; F, fungi) of microbial community between FPM and CPM.

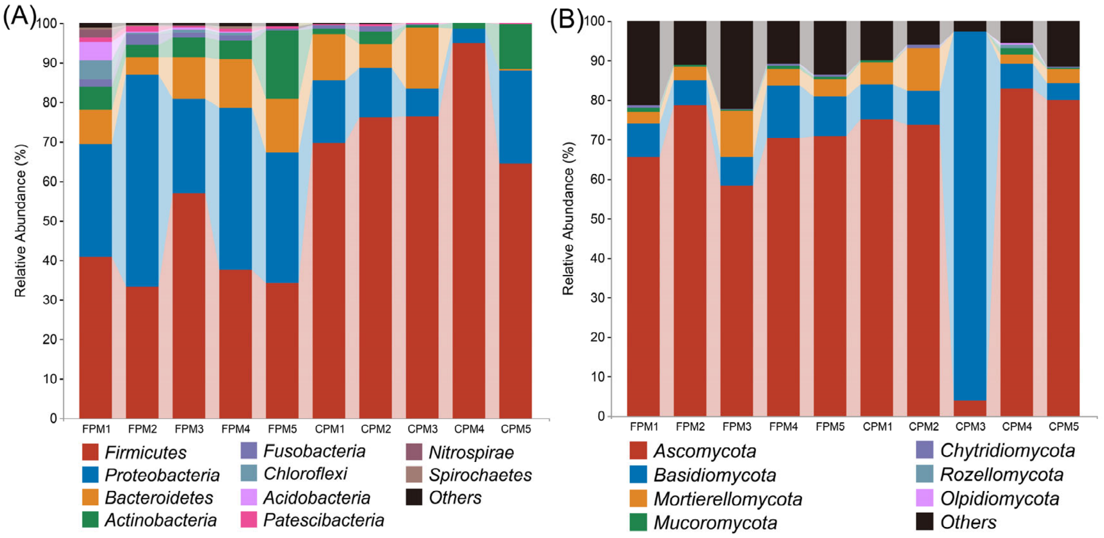

Figure S2 The composition of microbial community at the phylum level. (A) bacteria; (B) fungi.

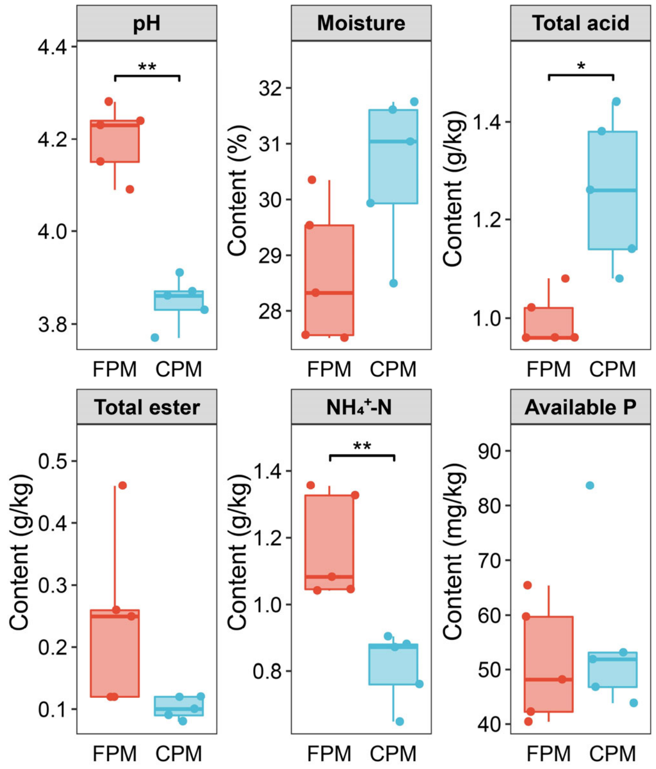

Figure S3 Variations in physicochemical parameters between FPM and CPM. Significant values based on *t*-test are shown as: *$p < 0.05$, **$p < 0.01$.

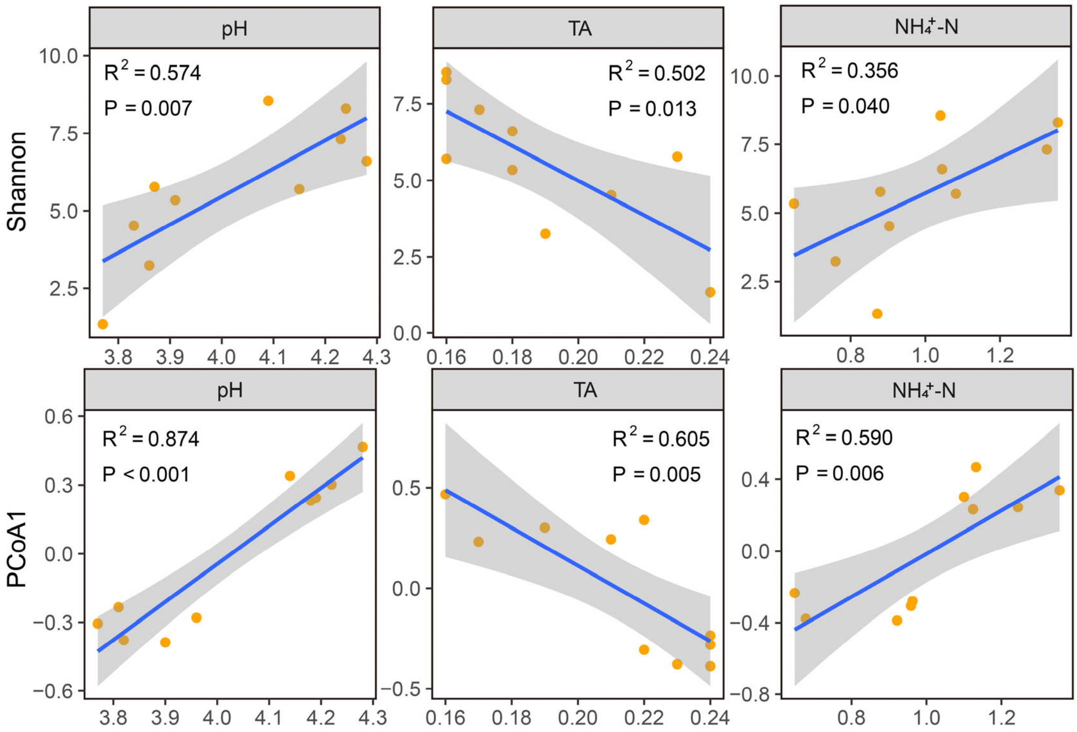

Figure S4 Linear regression analysis between potential driving factors and α/β-diversity of bacterial community.
